# Comment on ‘Hemispheric imbalance in mild cognitive impairment: a graph-theoretical analysis of multimodal brain networks’

**DOI:** 10.1080/07853890.2026.2618312

**Published:** 2026-02-05

**Authors:** Yuqi Liu, Jinyong Tian

**Affiliations:** a The First Clinical Medical College of Guizhou University of Traditional Chinese Medicine, Guiyang, PR China; b Department of Neuroelectrophysiology, Guizhou Provincial People’s Hospital, Guiyang, PR China

The study by Sun et al. presents an ambitious multimodal graph-theoretical analysis of hemispheric networks in Mild Cognitive Impairment (MCI) [1]. While the integration of functional, structural, and coupling metrics is commendable, several oversights critically undermine the interpretation of its core findings, particularly the conclusion of a specific ‘left-hemispheric disintegration’ in amnestic MCI (aMCI).

The significant defect lies in the conceptualization and statistical testing of ‘lateralization.’ The authors assess lateralization solely *via* within-group paired t-tests comparing left vs. right hemisphere metrics (e.g. Figure 3, 4). This approach identifies an *intra-group* asymmetry pattern but is statistically invalid for concluding that one group (e.g. aMCI) has *abnormal lateralization compared to others*. To claim abnormal lateralization, one must first calculate a lateralization index (LI) for each participant (e.g. (L–R)/(L + R)) and then perform *between-group comparisons* of these LI values [2]. The reported finding that ‘both MCI groups showed a significant leftward reduction in small-world properties’ (*p* < 0.05 within group) does not demonstrate that this leftward shift is different from controls. The healthy control (HC) group might exhibit a similar or even greater leftward bias, rendering the patient groups’ asymmetry unremarkable. This flaw invalidates the central theme of ‘hemispheric imbalance’ as a disease-specific signature.

The analysis of structure-function coupling (SFC) is methodologically problematic. The authors compute a single global Spearman correlation per hemisphere by vectorizing entire connectivity matrices. This approach discards all spatial information and is profoundly insensitive to region-specific coupling changes [3]. A loss of global SFC lateralization could result from opposing, localized increases and decreases in sub-networks, masking true pathophysiology. To support claims about network disintegration, a more informative approach would be to calculate node- or edge-specific coupling, perhaps within canonical cognitive networks.

Finally, the translational leap from these cross-sectional, group-average network metrics to specific neuromodulation targets (e.g. TMS to left TPOsup) is premature and unsupported. The study lacks any validation of these network features as *individual-level* biomarkers. The moderate correlations in Figure 8 (*r* ∼0.3-0.4) account for less than 20% of variance, indicating poor diagnostic or prognostic specificity. Recommending intervention strategies based on such associations ignores the well-established heterogeneity within MCI subtypes and the poor reproducibility of single-site neuroimaging biomarkers [4].

To salvage the valuable multimodal data, we propose: 1) Re-analysis using proper lateralization indices (LI) and between-group ANCOVA; 2) Employment of NBS for node/edge-wise comparisons with stricter family-wise error correction; 3) Investigation of SFC within predefined large-scale networks (e.g. Default Mode, Salience) rather than globally; and 4) Explicit acknowledgment that the observed effects are small, group-level phenomena requiring replication in an independent cohort before any mechanistic or clinical interpretation can be sustained [5].
